# The Diagnostic Accuracy of Pulse Oximetry for Assessing Pulp Vitality: A Systematic Review

**DOI:** 10.7759/cureus.76820

**Published:** 2025-01-02

**Authors:** Divya Gupta, Amar K Shaw, Ashwini Gaikwad, Pallavi Kale, Sneha M Deshpande, Arti Gachake

**Affiliations:** 1 Conservative Dentistry and Endodontics, MA Rangoonwala College of Dental Sciences &amp; Research Centre, Pune, IND; 2 Public Health Dentistry, Bharati Vidyapeeth (Deemed to be) University Dental College and Hospital, Pune, IND; 3 Conservative Dentistry and Endodontics, Bharati Vidyapeeth (Deemed to be) University Dental College and Hospital, Pune, IND; 4 Oral Pathology and Microbiology, Bharati Vidyapeeth (Deemed to be) University Dental College and Hospital, Navi Mumbai, IND; 5 Prosthodontics, Bharati Vidyapeeth (Deemed to be) University Dental College and Hospital, Pune, IND

**Keywords:** endodontics, pulp diagnosis, pulp vitality, pulse oximetry, systematic review

## Abstract

This review was performed to evaluate the diagnostic accuracy of pulse oximetry (PO) in patients with apical periodontitis (AP) or undergoing an endodontic treatment. Preferred Reporting Items for Systematic Reviews and Meta-Analysis-Diagnostic Test Accuracy (PRISMA-DTA) checklist was followed to carry out this review. The diagnostic potential of pulse oximetry for pulp vitality status was studied through databases like PubMed, Google Scholar, EBSCOhost from 2000 to 2024. Extraction or manual calculation of true-positive, false-positive, true-negative, false-negative, sensitivity, and specificity values were done. Quality assessment of diagnostic accuracy studies (QUADAS)-2 tool and RevMan ver. 5.3 qualitatively assessed the included studies. The included seven studies evaluated 654 teeth and reported a low risk of bias (ROB). The included studies evaluated the reliability, efficacy, and diagnostic ability of PO and compared it to other conventional pulp vitality and sensitivity tests like a cold test, thermal test, electric pulp test (EPT), spray, and rubber cups. Its overall sensitivity ranged from 81-100% (mean 95%) and overall specificity ranged from 94-100% (mean 99%). It was concluded that pulse oximetry overall is more reliable, superior, effective and has overall diagnostic efficacy in detecting the target condition compared to other conventional modalities.

## Introduction and background

Primary care in dentistry encompasses various aspects, including accurate diagnosis, treatment of symptomatic pathologies, disease prevention, and patient education [1]. Dental clinicians must prioritize accurate diagnosis, particularly for symptomatic patients presenting with pain [2].

Vitality assessment of teeth is crucial in dentistry and often gets complicated by the dental pulp's enclosure within calcified tissue [3,4]. Indirect methods are necessary due to the inability to directly inspect pulp tissue [5]. Pulp vitality relies on vascular supply and pulpal circulation [6,7]. Conventional pulp testing methods, such as thermal and electrical tests, have limitations as they measure neural responses rather than direct pulp vitality [8,9]. Blood circulation, and not the neural innervation, determines the pulp vitality [10,11].

Dentists face additional challenges with developing teeth and child patients' emotional and cognitive development [10]. Pain can lead to misdiagnosis, and various methods have been proposed to assess pulpal circulation [10]. Pulse oximetry, a non-invasive method, measures vascular health by evaluating oxygen saturation [11]. It is objective, painless, and sensitive in detecting pulpal blood components [12]. Reviews have shown promise in using pulse oximetry as a diagnostic tool for pulpal vitality [10,12,13]. This technique directly measures blood oxygen saturation levels, providing a reliable indicator of pulp status [14,15]. Unlike traditional pulp tests relying on patient subjective responses, pulse oximetry offers an objective assessment [12].

Up to now, no research has delivered a thorough, quantitative, and diagnostic accuracy evaluation of pulse oximetry (PO) for assessing pulp vitality and diagnosing pulp conditions. Consequently, we revised our investigation based on current scientific evidence and performed this review to evaluate the diagnostic accuracy of pulse oximetry relative to other pulp vitality or sensitivity assessments in patients needing endodontic care.

## Review

Protocol development

This systematic review was designed according to the Preferred Reporting Items for Systematic Reviews and Meta-Analysis-Diagnostic Test Accuracy (PRISMA-DTA) checklist [16]. It was registered in PROSPERO with the registration number CRD42020246543.

Study design

We formulated a focused research question according to the participants (P), index test (I), reference standard (R), and target condition (T) format as follows: "Does pulse oximetry (index test) have different diagnostic accuracy than other standard vitality tests (gold standard) for pulpal diagnosis?".

Search strategy

Keywords and Medical Subject Headings terms were selected and combined with Boolean operators such as AND/OR, and we conducted the search strategy according to the PIRT format (population, index test, reference standard, and outcome assessed; Table 1).

**Table 1:** PIRT format PIRT - population, index test, reference condition, and target condition; MeSH - Medical Subject Headings

Component	Strategy
Population	("endodontic treatment"[MeSH Terms] OR "apical periodontitis" OR "root canal treatment” OR ("mature AND open apex"[MeSH Terms] OR "dental pulp" OR ("treatment plan"[MeSH Terms] OR ("root development" OR ("pulp vitality"[MeSH Terms]
Index test	("vitality test"[MeSH Terms] OR "pulse oximetry" AND "oxygen saturation" AND "pulp vitality" OR "pulp sensitivity" OR "laser doppler flowmetry" OR "systemic oxygen saturation"[MeSH Terms] OR ("endodontics" AND "diagnostic efficacy")
Reference condition	("cold test" OR "thermal test"[MeSH Terms] OR "electric pulp test" AND "pulp vitality" OR "pulp sensitivity"
Target condition	("sensitivity” OR "specificity" OR "diagnostic odds ratio"[MeSH Terms] OR ("positive likelihood ratio" AND "negative likelihood ratio” OR "positive predictive value" OR ("negative predictive value" AND "summary receiver operating characteristics" AND "diagnostic accuracy")

We included studies published from January 2000 to April 2024 composed in the English language, and that are accessible as full-text articles for free. These studies were cross-sectional and analytical investigations assessing the diagnostic precision of pulse oximetry against other pulp vitality assessments. The studies included teeth with fully developed apices, free from caries, and large restorations without indications of necrosis or symptoms. These studies presented results related to sensitivity, specificity, accuracy, and predictive values (PV) through various methods regardless of the approaches used to measure the outcomes. We excluded in-vitro studies, animal studies, case reports, and case series. Research that does not present primary outcomes related to accuracy, sensitivity, and specificity, as well as instances where primary outcomes cannot be derived from the provided raw data, were not included. Studies should not include patients with systemic complications, intellectual disability, inability to respond to vitality tests, and teeth with calcific metamorphosis or with the presence of any pulp stones.

Screening process

Two authors conducted a rigorous two-phase screening process, initially evaluating titles and abstracts to remove irrelevant records and assessing full-text articles for eligibility. Disagreements were resolved through discussion and when necessary, a third reviewer was consulted to achieve consensus.

Data extraction

For each included study, we extracted descriptive data, including author, year of publication, sample size, design of the study, outcomes measured, parameters evaluated, and conclusions drawn. Metrics such as sensitivity and specificity were gathered, and data like true positive (TP), true negative (TN), false positive (FP), and false negative (FN) were computed for the studies using the formulas following formulas: a) False positive = (1-specificity) x (1- diseased cases/ total sample); b) True negative = specificity x (1- diseased cases/total sample); c) True positive = sensitivity x diseased cases/ total sample; d) False negative = (1- sensitivity) x diseased cases/total sample.

Assessment of methodological quality

To assess methodological quality, we used the quality assessment of diagnostic accuracy studies - 2 (QUADAS-2) tool [17], evaluating several domains like patient selection, index test, reference standard, and timing and flow of patients. Each domain included flagging questions with responses of "Yes," "No," or "Unclear." The overall risk of bias was assessed as high when answered 'No' to any question, low when responded 'Yes' to all inquiries, and unclear when replied 'Unclear' to all questions or combined with any 'Yes' within the Review Manager (RevMan) software version 5.3.

Results

**Figure 1:**
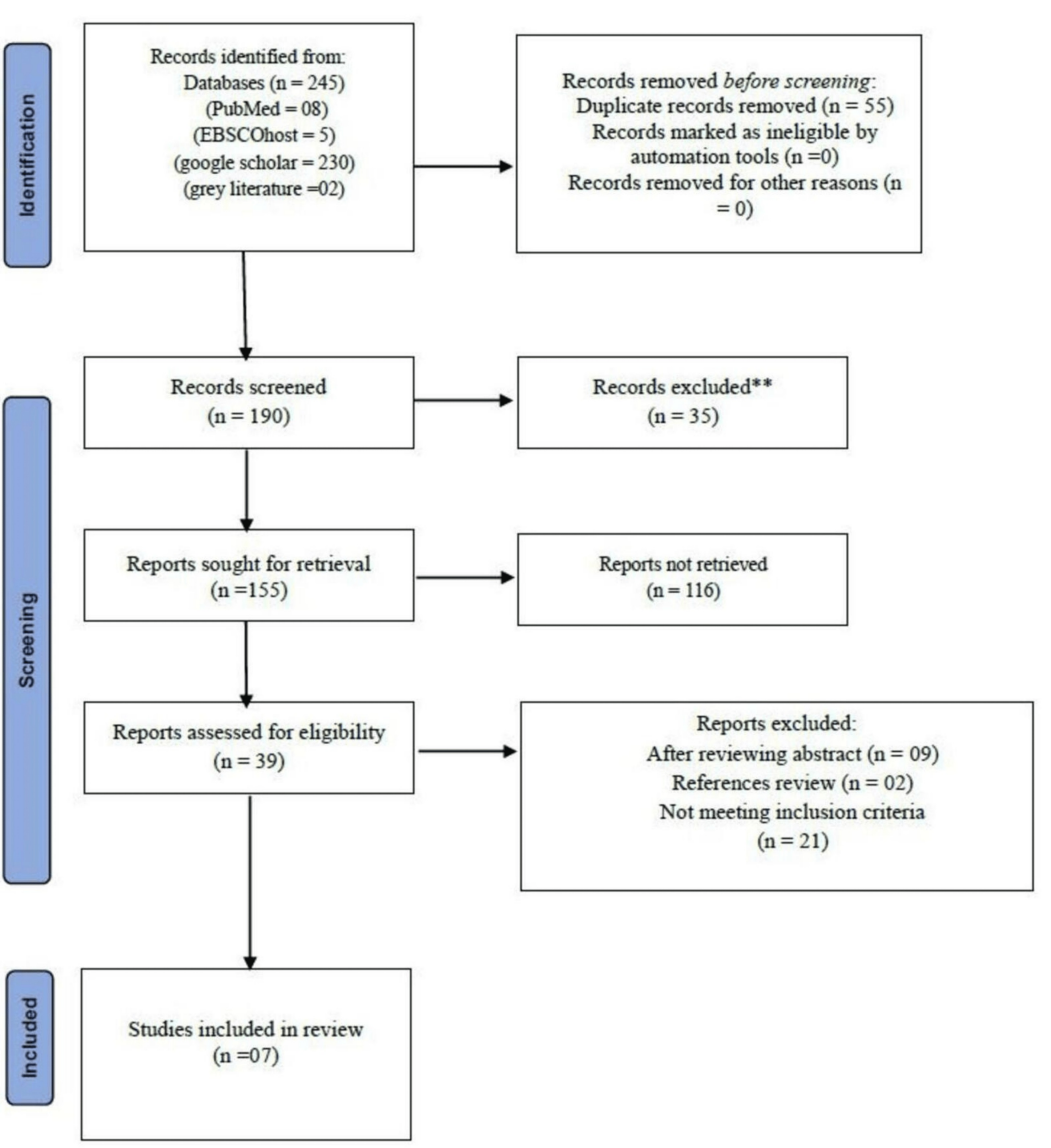
PRISMA 2020 flow diagram PRISMA - Preferred Reporting Items for Systematic Reviews and Meta-Analysis

A total of 155 studies were retrieved from the database search, and after removing duplicates and applying all inclusion and exclusion criteria, seven studies remained eligible for qualitative synthesis, as illustrated in Figure 1.

Study characteristics

As shown in Table 2, the data were drawn from seven studies with a combined sample size of 654 teeth (maxillary central and lateral incisors, mandibular premolars, mandibular first molars, and primary second molars) that received endodontic treatment, involving participants aged 7 to 18 years and employed a cross-sectional design [18-24]. The included studies were conducted in multiple countries, specifically two studies in Iran [18,19] and five studies in India [20-24]. All the included studies assessed, evaluated, and compared the reliability, efficacy, and diagnostic ability of pulse oximetry (PO) compared to other conventional pulp vitality and sensitivity tests (cold test, thermal test, electric pulp test (EPT), spray, rubber cups) for diagnosing pulp status. The result of these studies demonstrate that pulse oximetry is generally more reliable, superior, and effective, and demonstrates greater diagnostic efficacy in identifying the target condition than other traditional methods.

**Table 2:** Descriptive study details of included studies EPT - electric pulp test; PO - pulp oximetry; FN - false negative; FP - false positive; TN - true negative; TP - true positive; NPV - negative predictive value; PPV - positive predictive value

Author, year	Country	Sample size	Study design	Outcome assessed	Parameters evaluated	Conclusion
Karayilmaz et al., 2011 [18]	Iran	59 (12-18 years) (maxillary central and lateral incisors)	Cross-sectional study	To compare reliability of PO and EPT for diagnosing pulp status	Sensitivity, specificity, PPV, NPV	PO and EPT had equal diagnostic efficacy in diagnosing the target condition
Dastmalchi et al., 2012 [19]	Iran	24 (mandibular premolars)	Cross-sectional study	To compare the diagnostic ability of PO with other pulp vitality tests	Sensitivity, specificity, PPV, NPV	PO was superior to other vitality tests
Samuel et al., 2014 [20]	India	60 (7-18 years) (permanent maxillary central and lateral incisor)	Cross-sectional study	To compare accuracy of PO with conventional diagnostic methods (Cold test and EPT)	Sensitivity, specificity, PPV, NPV	PO was more reliable than conventional methods for accurately diagnosing pulp vitality in children
Shahi et al., 2015 [21]	India	155 (4-12 years) (primary 2^nd^ molar & permanent 1^st^ molar)	Cross-sectional study	To compare pulp vitality with PO and EPT	TP, TN, FP, FN	PO can be used as a reliable method for diagnosing pulp vitality
Janani et al., 2020 (a) [22]	India	37 (single root teeth)	Cross-sectional study	To evaluate diagnostic ability of PO in pulp status	Sensitivity, specificity, PPV, NPV, Accuracy	PO had highest diagnostic accuracy in various pulpal conditions
Janani et al., 2020 (b) [23]	India	79 (single rooted teeth)	Cross-sectional study	To evaluate diagnostic accuracy of PO compared to pulp sensibility tests (Heat test, cold test, EPT)	Sensitivity, specificity, PPV, NPV, Accuracy	PO was more reliable and accurate compared to other tests
Molassadolah et al., 2022 [24]	India	240 (Maxillary central and lateral incisor)	Cross-sectional study	To evaluate the diagnostic accuracy of PO in permanent teeth	Sensitivity, specificity, PPV, NPV, Accuracy	PO showed highest diagnostic accuracy and is more reliable and suitable method

As shown in below Table 3, pulse oximetry (index test) was compared with other pulp vitality tests. An overall sensitivity ranging from 81-100% and overall specificity ranging from 94-100% with a mean sensitivity and specificity of 95% and 99% was observed.

**Table 3:** Descriptive diagnostic accuracy values EPT - electric pulp test; PO - pulse oximetry

Author ,Year	Index Test	Comparator	True positive (TP)	True negative (TN)	False positive (FP)	False negative (FN)	Sensitivity (%)	Specificity (%)
Karayilmaz et al., 2011 [18]	PO	EPT	0.81	0.06	0.94	0.19	81	94
Dastmalchi et al., 2012 [19]	PO	EPT, cold spray, rubber cup	0.93	0	1	0.07	93	100
Samuel et al., 2014 [20]	PO	Cold test, EPT	1	0	1	0	100	100
Shahi et al., 2015 [21]	PO	EPT	0.31	0.31	0.32	0	98	100
Janani et al., 2020 (a) [22]	PO	Sensor holder, thermal test, EPT	1	0	1	0	100	100
Janani et al., 2020 (b) [23]	PO	Heat test, cold test, EPT	0.97	0	1	0.03	97	100
Molassadolah et al., 2022 [24]	PO	Cold test, EPT	0.95	0	1	0.05	95	100

Quality assessment

**Figure 2:**
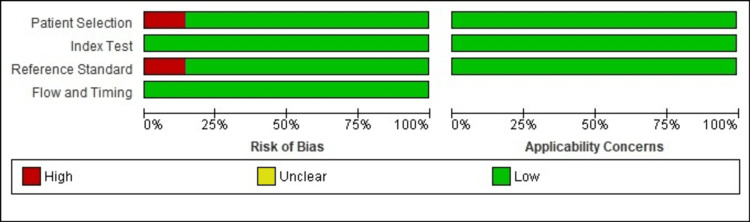
Cochrane risk of bias as percentages across all included studies

**Figure 3:**
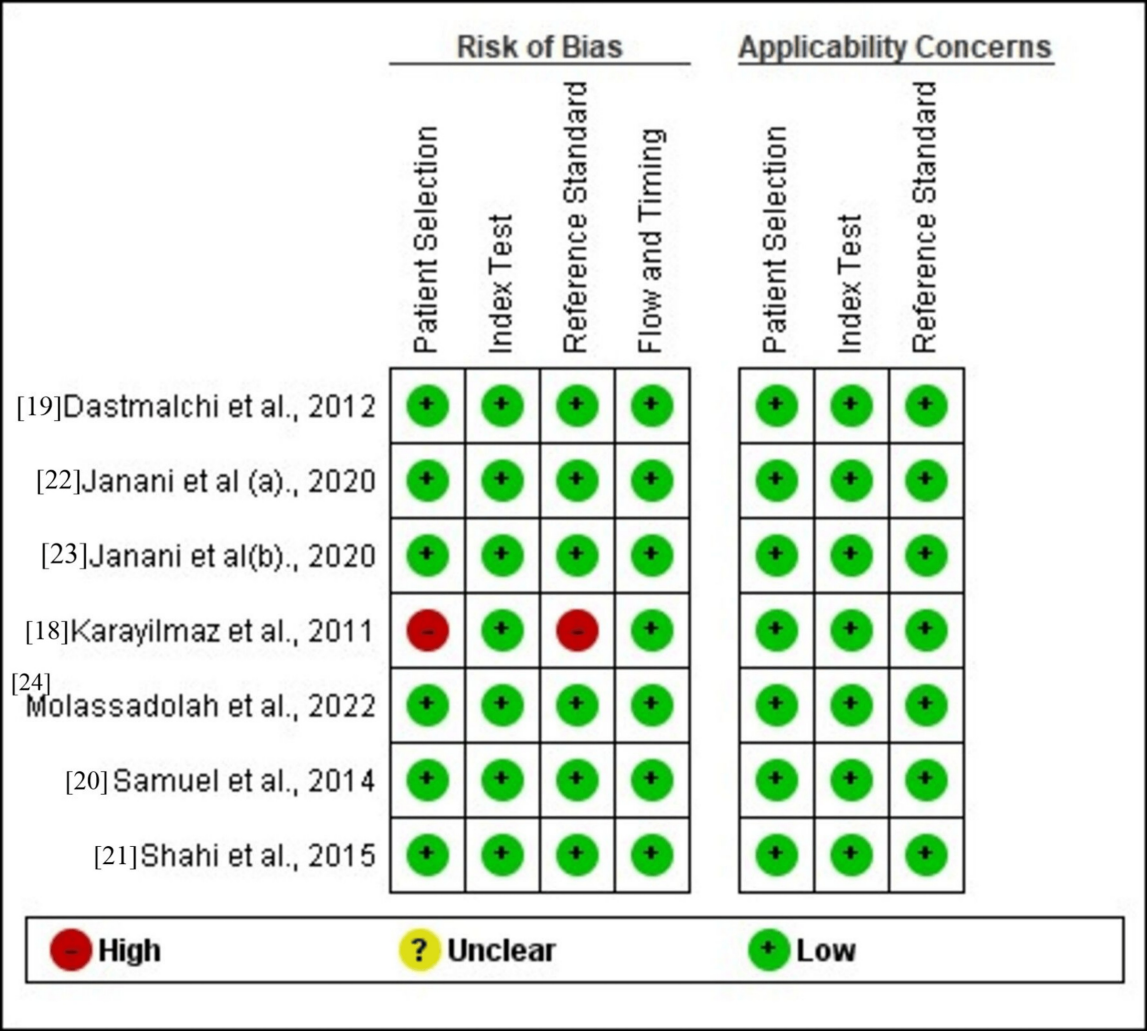
Cochrane risk of bias summary for each study

The index test, along with the flow and timing domain, exhibited a low risk of bias and applicability issues. In contrast, patient selection and reference standards faced a high risk of bias in one study [18], mainly due to varied patient enrollment methods, study design types, and inappropriate exclusion practices, as illustrated in Figures 2 and 3.

Discussion

Mainkar et al. [25] performed a systematic review to evaluate and compare the overall diagnostic precision of the cold pulp test (CPT), heat pulp test (HPT), electric pulp testing (EPT), laser doppler flowmetry (LDF), and pulse oximetry (PO). Databases were examined from January 1964 to December 2016 for research assessing the diagnostic effectiveness of these five dental pulp tests, providing outcomes related to sensitivity, specificity, positive predictive value (PPV), and negative predictive value (NPV). Data concerning true positive (TP), true negative (TN), false positive (FP), and false negative (FN) were gathered from every study. A total of 28 studies were incorporated into the review. A combined sensitivity of 0.87, 0.72, 0.78, 0.98, and 0.97 was found for CPT, EPT, HPT, LDF, and PO, along with a combined specificity of 0.84, 0.93, 0.67, 0.95, and 0.95, respectively. An overall precision of 0.84, 0.82, 0.72, 0.97, and 0.97 was observed. They concluded that LDF and PO demonstrated the highest diagnostic accuracy.

Lima et al. [26] performed a systematic review to assess and evaluate the effectiveness of pulse oximetry (PO), laser Doppler flowmetry (LDF), and ultrasound Doppler flowmetry (UDF) in diagnosing pulp conditions of injured teeth relative to other sensitivity assessments such as cold and electric testing. Databases were explored until May 2018. The final analysis included five studies. Because of the significant heterogeneity, establishing a comprehensive diagnostic accuracy for these vitality tests proved to be impossible.

Almudever-Garcia et al. [27] conducted a systematic review to assess and analyze the efficacy of pulse oximetry (PO) in determining pulp vitality. Databases were searched for studies comparing the diagnostic accuracy of PO to cold tests. From the results of the study, it was found that PO overall showed high sensitivity and specificity and a good potential for diagnosing pulp vitality.

Patro et al. [28] conducted a systematic review and meta-analysis to evaluate the diagnostic accuracy of different pulp sensibility tests such as pulse oximetry (PO), electric pulp tester (EPT), cold test (CT), and heat tests in determining pulpal health. They evaluated 10 studies qualitatively and five studies quantitatively. A pooled odds ratio of 628.5, 10.75, 17.24, and 3.27 was noted. Pairwise comparison demonstrated a generally high sensitivity and specificity for PO in relation to other tests. Additionally, the summary receiver operating characteristics (SROC) indicated that PO exhibited a generally high diagnostic accuracy. It was found that PO may be regarded as a test with the greatest diagnostic accuracy in comparison to other tests.

Saikiran et al. [29] conducted a meta-analysis to identify the average oxygen saturation levels (SpO2) in primary teeth using pulse oximetry. Databases were examined from January 1990 to January 2022 for research documenting the sample size and average SpO2 values. A total of five studies were part of the review, with three studies included for meta-analysis. It was found that the average fixed-effect measure of oxygen saturation in the pulp of primary teeth was 88.45% (95% CI 83.97 - 92.93%).

These systematic reviews and meta-analyses [25-29] data heterogeneity often hindered the formation of comprehensive qualitative and quantitative comparisons between the different pulp vitality tests. To the best of our knowledge, this is the first systematic review and meta-analysis to examine and determine the overall diagnostic ability of pulp oximetry while adhering to strict methodological standards.

Databases were searched from January 2000 to May 2024 for RCTs, comparative, and cross-sectional studies comparing pulse oximetry with other pulp vitality tests for pulpal diagnosis. Seven studies were included for qualitative synthesis [18-24]. Included studies reported the presence of a low risk of bias overall, and an overall sensitivity ranging from 81-100% and overall specificity ranging from 94-100% with a mean sensitivity and specificity of 95% and 99% was found suggesting that pulse oximetry overall had moderate to good ability in diagnosing the target condition. Furthermore, standardized diagnostic accuracy studies with strict reporting using standards for reporting of diagnostic accuracy studies (STARD) guidelines or longitudinal studies should be carried out to validate the study findings. The systematic review followed PRISMA guidelines, and the use of the QUADAS-2 tool for ROB assessment ensured robust methodology and transparent reporting.

Systematic reviews and meta-analyses represent the highest level of evidence in evidence-based practice because they provide transparent, reproducible methodologies and synthesize findings from multiple studies. However, the quality and heterogeneity of included studies can influence the strength and applicability of the evidence. Although our review included studies characterized by brief observation periods and known risk of bias, the overall quality of the included studies was sufficient to provide meaningful insights into the diagnostic ability of pulse oximetry.

## Conclusions

This systematic meta-analysis examined whether PO is an alternative to conventional pulp vitality tests for the diagnosis of pulp vitality. We investigated sensitivity, specificity, positive predictive value, and negative predictive value. It was concluded that pulse oximetry is more reliable, superior, effective, and efficacious in detecting the target condition compared to other conventional modalities. This review has managed to highlight the validity and overall diagnostic accuracy of pulse oximetry as a pulp vitality test for pulpal diagnosis.
